# Texture analysis and artificial neural networks for identification of cereals—case study: wheat, barley and rape seeds

**DOI:** 10.1038/s41598-022-23838-x

**Published:** 2022-11-11

**Authors:** Ł. Gierz, K. Przybył

**Affiliations:** 1 Institute of Machine Design, Faculty of Mechanical Engineering, Poznan University of Technology, ul. Piotrowo 3, 60-965 Poznan, Poland; 2 Department of Dairy and Process Engineering, Poznan University of Life Sciences, Food Sciences and Nutrition, Wojska Polskiego 31, 60-624 Poznan, Poland

**Keywords:** Engineering, Mechanical engineering, Physics, Applied physics, Information theory and computation

## Abstract

The scope of the research comprises an analysis and evaluation of samples of rape, barley and wheat seeds. The experiments were carried out using the author’s original research object. The air flow velocities to transport seeds, were set at 15, 20 and 25 m s^−1^. A database consisting of images was created, which allowed to determine 3 classes of kernels on the basis of 6 research variants, including their transportation way via pipe and the speed of sowing. The process of creating neural models was based on multilayer perceptron networks (MLPN) in Statistica (machine learning). It should be added that the use of MLPN also allowed identification of rape seeds, wheat seeds and barley seeds transported via pipe II at 20 m s^−1^, for which the lowest RMS was 0.05 and the coefficient of classification accuracy was 0.94.

## Introduction

One of the key agrotechnical treatments used for cereals and other cultivated plants determining their growth and satisfying crop is sowing^1^. In Poland, sowing is carried out with the use of mechanical and pneumatic sowers with working widths greater than 3 m. The development of sustainable agriculture in Europe and the world causes that agrotechnical processes, including sowing, are carried out more and more often by machines with huge working widths from 4 to 6 m or even up to 9 or 12 m^1^ with foldable frame systems^2–4^. Currently, sowers and cultivation and sowing aggregates are the most commonly used sowing machines^5^. By applying integrated soil cultivation^6^ one can come across the problems related to quality control and sowing breaks caused by a coulter blockage^7^ (blocked by wet soil or straw), which in turn may lead to blockage of the sowing pipe. The foldable frames of the sowers require application of pneumatic systems to transport the sowing material to the coulters.

In view of the above, machine operators face problems with installing pneumatical sowers, caused by insufficient evenness of seed division into separate rows^1,8–12^. This problem can lead to seed transport delays and difficulties to control sowing in terms of detecting sowing brakes caused by blocked feed pipes (transporting seeds).

In recent years, one can observe the development of various sowing control systems, mainly those based on photoelectric sensors capable of detecting individual seeds. One of the most popular solutions devised by the Vaderstad company is a system known under the commercial name of “seed eye”^13^. So far it has not been widely used for controlling the movement of sowing material in mechanical or pneumatic sowers^14^. The available structures, do not commonly use solutions such as placing sensors for blockages detection or sowing material counting at the end of a sowing pipe or in a coulter. In addition, no research results have been found to provide new techniques for detecting kernels in sowing material in order to count them and detect blockage of sowing pipes.

However, in specialized literature one can find examples of application of optical sensors used as markers, which allow fast, non-destructive and reliable identification of the best doses of gamma radiation in order to stimulate the parameters of soya growth^15^. What is more, one can also find research focusing on perceptibility of those sensors^16^. There are also systems with piezoelectric sensors inside the sower distribution head^17^ and on the outlet stub pipes^18^ as well as cases of using capacity sensors U. S. Patent 4782282 A. Unfortunately, insufficient capacity changes possible with this solution, indicate poor future perspectives for this method. In view of the above, it seems justified to search for online solutions that will enable to detect blockages as well as count seeds that are sowed, that can eliminate the necessity of carrying the so called calibration test. It will allow efficient calibration of the sowing unit both in fertilizers and seed sowers and other cultivation devices^19^.

Lack of a cheap sowing control system of cereal kernels to indicate blocked pipes results in occurrence of sowing gaps, (breaks) and subsequently leaves some parts of acreage unsown (dominated by weeds). Application of secondary sowing in the unsown area is possible but expensive, and this solution is not used by farm owners in Poland or in Europe^20^.

The aim of this study is to determine the velocity of kernels to be used for defining technical conditions and forces occurring when a kernel collides into the sensor, a bar equipped with a control system designed for the needs of the sensor construction, or a control bar used in mechanical and pneumatic sower units.

In standard multiple row sowers and automated sowers^21,22^ for sowing corn and other cultivated plants, the above mentioned system could be applied in order to control blockages of sowing pipes. The potential of the above solution can be utilized for detecting blockages in pipes transporting seeds and fertilizers according to a method called strip-till. In order to provide a correct study of such a system it is crucial to obtain all necessary technical data to construct the sensor and the whole unit. The first step is to determine the velocity of kernels in order to estimate forces occurring when a kernel collides into a sensor or a control bar. Until now, there has been no database of velocity distribution of kernels in the sowing pipe outlet (air and seed), or a method determining velocity of objects (kernels).

Currently, there are attempts to find solutions, that will allow to control the velocity of objects (seeds) in real time.

In this study, the authors have used one of the artificial intelligence methods, i.e. artificial neural networks ANN whose functioning involves proper using of mathematical or programing formulas^23,24^. Artificial neural networks are most often used for data with no ordered or simple computing structure^25,26^. ANNs are mostly used in information including processing of image (bitmap)^27–29^, sound (acoustic wave)^30,31^ and text (numerical data)^32–36^. The simplest form of a neural model consists of layers of neurons, in which a neuron from one layer generates a signal constituting one of the arguments for each neuron of another layer.

A decision was made to examine the velocity of two medium size seeds (wheat and barley) and one fine-grained seed (rape). It should be added that Multi-Layer Perceptron (MLP) neural networks were used in order to detect and count the number of kernels in the sowing material for the most popular varieties of cereals cultivated in Poland. The practical goal was to develop neural networks capable of fast identification of various types of cereal kernels. The use of an ultra-high-speed camera to register images for the needs of designing new sensors and sowing control systems can be viewed as an innovative approach.

## Materials and methods

### Preparation of samples

It should be said that for the needs of the research, the authors used varieties of cereals that are most commonly sown in Poland. It is also worth noting that, based on statistical data from 2019, including the last ten years (https://www.fao.org/faostat/en), Poland is ranked seventh in the global ranking of rape production. The research material consisted of winter wheat seeds Elixer with the initial moisture level of 9.8%, winter barley seeds LG Veronika with the initial moisture level of 12.2% and winter rape seeds Kite with the initial moisture level of 9.0%. All the research material came from the Main Seed Warehouse Top Farms Seeds, Production Plant in Runów located in Greater Poland Province. Based on the announcement of the Marshal of the Sejm of the Republic of Poland about the Legal Protection of Plant Varieties of January 22, 2021 (Journal of Laws of 2021, item 213) and the breeder's declaration that the indicated varieties: Kite (rape), LG Veronika (barley) and Elixer (wheat) are protected by law by the breeder; the authors have received this permission. The breeder agreed to provide the above-mentioned plant material, which complies with the national guidelines of the Main Seed Warehouse Top Farms Seeds, a production plant in Runów, located in the Greater Poland Province. With the breeder's consent, the authors were allowed to use their plant material only for the purposes of scientific research, including carrying out tests of e.g. seeding simulation.

Prior to the experiment, the sowing dose (ration) was determined for each variety of cereals. The sowing dose (ration) was determined based on the most commonly used dose in Poland. For the needs of the research, a dose (ration) of 115 kg ha^−1^ and 185 kg ha^−1^ was established for wheat, a dose of 170 kg ha^−1^ was established for barley and a dose of 4.4 kg ha^−1^ was established for rape, respectively. A dose of 115 kg ha^−1^ is used for wheat seeds in fertile soils, whereas a dose of 185 kg ha^−1^ is used in medium fertile soils. It should be added that all kernels were properly selected prior to the research.

Next stage of the research involved adapting the stream of kernel mass transported via pneumatic pipe for selected structures: I and II. To check a possibility of controlling the sowing material movement in the sower elements, irrespectively of the seed dose, two dose levels were examined for wheat kernels, i.e. 280 seeds/m^2^ and 450 seeds/m^2^ (structure I and II). In the case of barley and rape seeds, only one dose level was used. In terms of barley, sowing density was set at the level of 375 seeds/m^2^ (structure I and II). For rape, sowing density was set at the level of 80 seeds/m^2^ (structure I and II).

### Test stand

**Figure 1:**
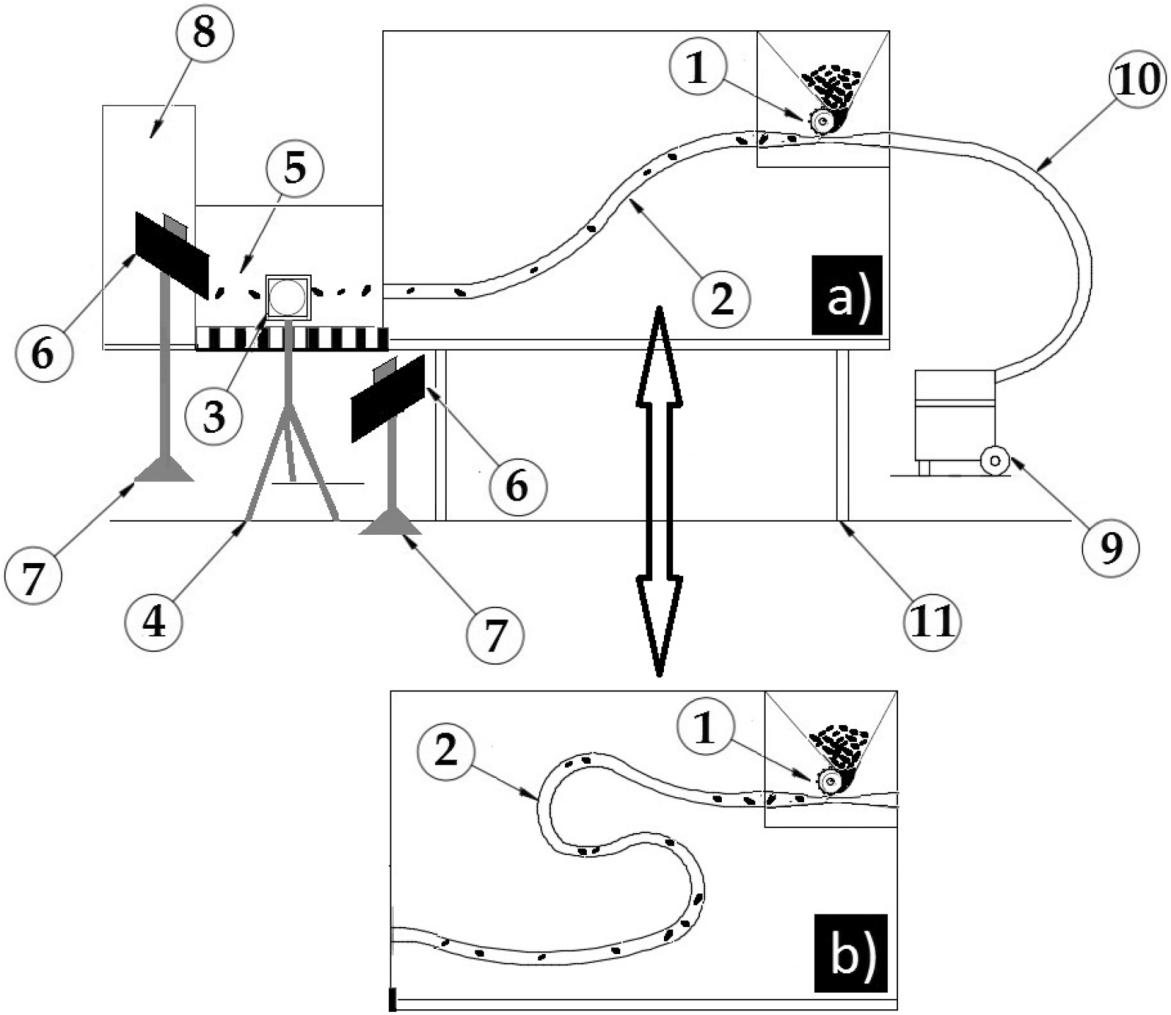
The scheme of the test stand by Gierz et al.^31^ (**a**) pipe configuration I, (**b**) pipe configuration II,, 1- drilling unit (a feeder), 2-seed pipe, 3- high-speed camera, 4- adjustable camera stand, 5—Screen with scale , 6- LED lamps with stabilization system, 7- adjustable lamp stand, 8- seed container, 9-a vacuum cleaner with a blower function, 10- air pipe, 11- load-bearing construction of the stand.

In order to provide the seed blockage control system with real working conditions, the author’s original research stand was constructed, which consisted of (Fig. 1):

- Sowing unit consisting of seed crate, sowing rollers driven by electric engine with adjustable rotation speed (2a, 2b),
- Air and seed pipe with configuration I (2a),
- Air and seed pipe with configuration II (2b),
- Chronos camera 1.4. (3a),
- A tripod camera stands with adjustable height (4a),
- Screen with scale (10 mm) (5a),
- Two LED lamps with stabilization system (6a),
- Lamp stand with adjustable height (7a),
- Seed container (8a).
- Vacuum cleaner with blower and rotation regulation (9a),
- Air pipe (10a),
- Load-bearing construction of the stand (11a).

Like in the previous research^37^, velocity of air stream transporting kernel in a pneumatic tube was 15, 20, 25 m s^−1^ (those values of air stream velocities are typically used in pneumatic sowers). It should be observed that various configurations of the pneumatic tube arrangements and lengths are used in pneumatic sowers. With reference to the earlier research^37^, it was decided to compare the two most common seed-air tube configurations. The first of them (a) in the shape of extended “S” letter with the tube length of 1.5 m. and the second (b) in the shape of tightened “S” letter with the tube length of 2.0 m.

The kernel dispenser was devised and built according to the pattern of a standard dispenser used in sowers, which was described in an earlier experiment^37^.

The tests involved providing the dispenser with proper rotation velocity in order to obtain sowing density, that is, 280 grains of wheat/m2 and 450 grains of wheat/m2 respectively, 375 grains of barley/m2 and 80 grains of rapeseed/m2.

As part of the control process of kernel transporting air stream velocity, A pressure Anemometer called VOLTCRAFT VPT−100 was used . The measuring range of the device is 1 to 80 m s^−1^ and measuring accuracy + /− 2.5%. Air stream transporting seeds in a seed-air tube was generated by vacuum STANLEY SXVC20PTE with rated power of 1200 W, which has also the function of blower. The air stream velocity within the range from 5 to 50 m s^−1^ was regulated with potentiometer, which controlled the vacuum engine speed.

**Figure 2:**
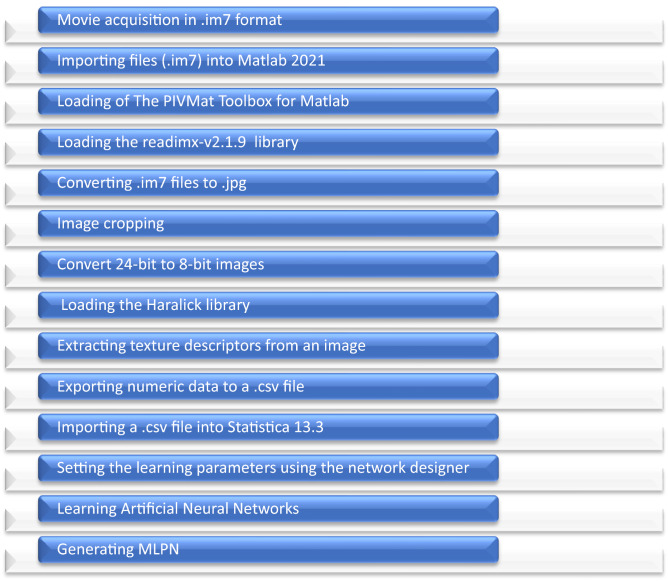
The scheme of image processing and creation of Artificial Neural Networks.
- Variable:
  Velocity of air stream: 15 m s^−1^, 20 m s^−1^, 25 m s^−1^,
  Grain variety: wheat, barley, rapeseed
  Dose of seeds to be sown: wheat 359.0 g min^−1^ , which corresponds to 115.0 kg ha^−1^ and 575.65 g min^−1^, which corresponds to 185.0 kg ha^−1^
- Result factors:
  Kernel velocity m s^−1^,
  Kernel acceleration m s^-2^.

The following factors were accepted for the needs of this study:

- Fixed:
  Dose of seeds to be sown: barley 526.50 g min^−1^, which corresponds to 170.0 kg ha^−1^ and rapeseed 13.85 g min^−1^, which corresponds to 4.4 kg ha^−1^;
  Sowing velocity: 15 km h^−1^,
  Tube configuration: (a) and (b) (Fig. 2),

### Preparation of research material with camera

Graphic data of wheat, barley and rapeseed with different variables (velocity and grain variety, configuration of seed-air tube) was acquired using a high-speed camera HighSpeedStar5, which is characterized by high image projection frequency. This device is equipped with an image converter of CMOS type. This fast device enables taking videos at 3000 frames per second, with the resolution of 1024 × 1024 and taking photos at 30,000 photos per second with the resolution of 256 × 256.

As a result, 18 videos in .im7 format were taken, for which data recording speed was about 9400 kb/s with the image resolution of 640 × 360. Six research variants were created in order to develop learning sets, and the next step was to design neural networks for them. Each research variant consisted of 3 videos with 3 classes of grain varieties (wheat, barley, rapeseed). The same parameters of image projection were used, i.e. 58 frames per second, to provide measuring repeatability of the results and compare velocities.

### Image processing in MATLAB

At the first stage of the research, image conversion was carried out to obtain a 24-bitmap image with 1437 × 1253 resolution in .jpg format (Fig. 2). It should be added that the above conversion applied to each class (grain variety) consisting of 212 images in .im7 format. In order to do that the author used software called MATLAB 2021b, which enabled image conversion from .jm7 to .jpg. To acquire image in .jpg format, the author used software called The PIVMat Toolbox for MATLAB by Frederic Moisy (https://www.mathworks.com) and library readimx-v2.1.9 (https://www.lavision.de). PIVMat Toolbox contains a set of functions enabling to import images in .im7 format. Library readimx allowed to read a given file format effectively with MATLAB. Another step was to crop the 24-bitmap 1437 × 1253 resolution images in jpg format to 931 × 931 resolution images.

The next stage was to process the images so as to highlight characteristics of the texture. The texture is used to interpret the image details^33,38,39^, it carries information about its surface, color and other parameters related to the lighting model including: color of reflected and stray light, degree of transparency and light refraction coefficient. An analysis of the texture was carried out using a grey-level co-occurrence matrix (GLCM)^27,40^. In order to apply GLCM to present details of the surface with procedural textures, it was necessary to carry out image conversion from 24-bitmap to 8-bitmap image depth in MATLAB. The prepared database consisting of 8-bitmap images was imported again to MATLAB in order to isolate 20 descriptors of texture from Haralick library^41–44^: contrast (contr)^45^, correlation (corrm), cluster prominence (cprom), cluster shade (cshad), dissimilarity (dissi), angular second moment (energ)^37^, entropy (entro)^37^, homogeneity (homom), homop, maxiumum probability (maxpr), sum of square variance (sosvh), sum of average (savgh), sum of variance (svarh), sum of entropy (senth), difference variance (dvarh), difference entropy (denth), info. measure of correlation 1 (inf1h), info. measure of correlation 2 (inf21h), inverse difference normalized (indnc), inverse difference moment (idmnc).

### Multilayer perceptron networks

**Figure 3:**
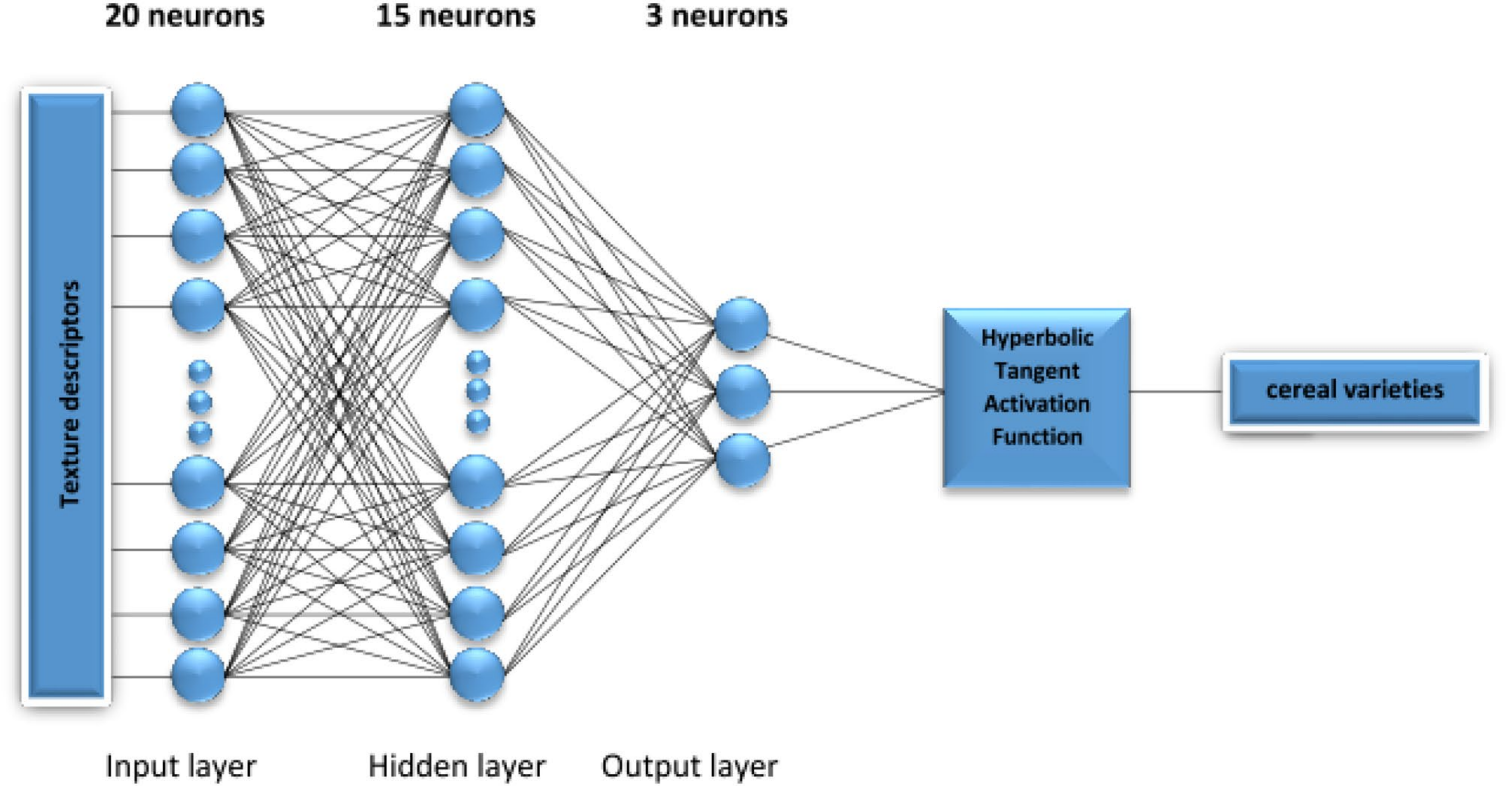
Artificial Neural Network used for the experimental data.

The research included the process of network machine learning. Multi-Layer Perceptron (MLP) neural networks were designed. Each structure of Multi-Layer Perceptron Layer Network (MLPN) consisted of 20 neurons in the input layer, 15 neurons in the hidden layer and 3 neurons in the output layer (Fig. 3). The input layer of MLPN defined 20 texture descriptors and determined classes of kernels (variety of grain). Each class of the learning set included 212 learning cases. In the end, the set consisted of 636 cases (Table 2). Six neural networks were prepared for the comparison process to assess the effectiveness of velocity distribution recognition for the selected classes of kernels. The configurations of networks with different kernel transporting air stream velocities (15 m s^−1^, 20 m s^−1^, 25 m s^−1^) and type of the seed-air tube (p1, p2) are presented in Table 1.

**Table 1:** Configuration of Artificial Neural Networks based on air velocity and position of the seminal-air tube.

Type	15 [m s^−1^]	20 [m s^−1^]	25 [m s^−1^]
p1	MLP-p1_15	MLP-p1_20	MLP-p1_25
p2	MLP-p2_15	MLP-p2_20	MLP-p2_25

### Statistical analysis

A statistical analysis was carried out for individual texture descriptors, and Tukey test was carried out for p value of 0.05. It should be added that Statistica 13.3 software was applied in order to carry out a statistical analysis.

## Results and discussion

### Machine learning

According to the research on determination of the number of the network hidden layers, 15 hidden layers were identified. After aggregation of the input data including weights, it was also necessary to determine the summary signal of stimulation. In order to obtain high effectiveness of the kernel class recognition, a hyperbolic tangent was selected as an activation function for neurons in the output layer. Wag reduction was also applied for neurons in the output layer. 1000 epochs were determined in order to train the network. Determining the number of epochs allows to present all cases for a given network, one after another. The output values will be compared with the assumed values by determining an error value. A simulation of the designed networks was carried out in Statistica v.13.3. software. In the process of learning, an adequate neural model was selected for each research variant, which was characterized by the highest classification capability. The research results obtained for the remaining networks are presented in Table 2. It should be added that Broyden–Fletcher-Goldfarb–Shanno (BFGS)^46^ algorithm was used in the prepared networks like in the case of the research on recognizing a raspberry powder carrier. According to the research results, BFGS algorithm becomes quite effective for the functions used in the author’s previous original research^33^. The lowest Root Mean Square Error (RMSE)^47^ at the level of 0.050 for the sets: training, testing and validation was reached by MLP-p2_20 with the seed-air tube type II. The lowest RMSE at the level of 0.060 was reached by MLP-p1_15 with the seed-air tube of type 1. The research on triticale^37^ indicates that the use of the seed-air time of type 1 along with an increase in the velocity of kernel transporting air stream (rapeseed, wheat and barley), leads to an observable increase in the network error, which in turn leads to deterioration of classification effectiveness. Like in the previous research, classification accuracy of most results (Table 2) achieved for the tested set was above 0.90^37^. It is worth highlighting that in the case of wheat kernels, barley kernels and rapeseed kernels, classification accuracy was found to be excellent for the air stream flow level of 15 and 20 m s^−1^ in the seed-air tube type 1.

**Table 2:** Results of training process Artificial Neural Networks.

Name ANN	MLP-p1_15	MLP-p1_20	MLP-p1_25	MLP-p2_15	MLP-p2_20	MLP-p2_25
Structure MLPN	20-15-3	20–15-3	20-15-3	20-15-3	20-15-3	20-15-3
Training error	0.054	0.085	0.124	0.045	0.040	0.096
Validation error	0.063	0.095	0.245	0.158	0.063	0.126
Testing error	0.054	0.074	0.149	0.137	0.084	0.095
Quality of learning	0.946	0.915	0.876	0.955	0.960	0.904
Quality of validation	0.937	0.905	0.755	0.821	0.937	0.874
Quality of testing	0.941	0.926	0.851	0.863	0.916	0.905
RMSE	0.060	0.085	0.146	0.079	0.050	0.101
Accuracy	0.941	0.915	0.827	0.880	0.938	0.894
Learning cases	636	636	636	636	636	636
Activation function in output layer	Exp/Tanh	Log/Tanh	Tanh/Tanh	Tanh/Tanh	Tanh/Tanh	Tanh/Tanh
Training algorithm	BFGS 269	BFGS 89	BFGS	BFGS 352	BFGS 349	BFGS 111

One of the difficulties to be coped with is distinguishing between the classes of kernels. It results from the fact that grains of different classes differ in sizes. The above translated into the results of network training, taking into consideration MLP-p1_25 and MLP-p2_25, for which RMSE reached 0.146 and 0.10, respectively. Unfortunately, these results are less satisfying. However, it should be said that the highest effectiveness in cereal kernel recognition was achieved for the seed-air tube type 2 (MLP-p2_20). In this solution the classification coefficient for air stream at the working level of 20 m s^−1^ was 0.92.

As already mentioned, when designing each network variant, the same parameters were determined, i.e. 20 texture descriptors, number of epochs, number of hidden layers and the output layer activation function. As a result, high effectiveness was obtained for 3 network variants. It turns out that the optimal solution for cereal kernel image recognition can be obtained with air stream velocity at the level of 20 m s^−1^, for the proposed configuration I and II of the seed-air tubes.

### Statistical analysis

Tables 3, 4, 5, 6, 7, 8 present an analysis of variants (ANOVA) of 20 texture descriptors for 9 kernel classes, which differ in terms of the seed-air tube structure used. The analysis allowed to compare different classes of cereal kernels on the basis of texture descriptors (indirectly acquired from digital images). When analyzing variables Contr, Corrm, Cprom, Dissi, Entro, Senth, Dvarh, Denth and inf21h, one can observe a similarity between the classes of wheat kernels transported with the air stream velocity at the level of 15 m s^-1^ (Table 3) and 20 m s^−1^ (Table 4) via the seed-air time of type 1. In the case of variables such as Cshad, Energ, Homom, Homop, Maxpr, Sosvh, Svarh, Savgh, inf1h, Indnc and Idmnc, an analysis of variance showed similarity between the classes of barley for the seed-air tube of type 1, transporting kernels with the velocity of air stream at the level of 20 m s^-1^ (Table 4) and 25 m s^−1^ (Table 5). By carrying out a statistical comparison of texture descriptors, one can observe similarities in the research groups containing medium size kernels of wheat and barley.

**Table 3:** Texture analysis of kernels while measuring selected air stream velocities (15 m s^−1^) and the position of the seed-air tube type I.

	**contr**	**corrm**	**cprom**
wheat_p1_15	1.792 ± 0.105 a	0.451 ± 0.010 a	261.146 ± 25.388 a
barley_p1_15	2.264 ± 0.106 d	0.485 ± 0.009 c	370.350 ± 29.510 d
rapeseed_p1_15	1.997 ± 0.110 b	0.464 ± 0.009 b	304.604 ± 27.348 b
	**cshad**	**dissi**	**energ**
wheat_p1_15	− 27.234 ± 2.274 e	0.634 ± 0.032 a	0.440 ± 0.022 e
barley_p1_15	− 36.848 ± 2.466 b	0.775 ± 0.030 d	0.345 ± 0.019 b
rapeseed_p1_15	− 31.120 ± 2.375 d	0.698 ± 0.033 b	0.395 ± 0.022 d
	**entro**	homom	homop
wheat_p1_15	1.692 ± 0.073 a	0.809 ± 0.009 e	0.810 ± 0.783 e
barley_p1_15	2.014 ± 0.069 d	0.770 ± 0.008 b	0.772 ± 0.739 b
rapeseed_p1_15	1.838 ± 0.073 b	0.790 ± 0.009 d	0.792 ± 0.762 d
	**maxpr**	**sosvh**	**savgh**
wheat_p1_15	0.657 ± 0.017 e	56.501 ± 0.495 e	14.845 ± 0.083 e
barley_p1_15	0.578 ± 0.017 b	54.117 ± 0.566 b	14.441 ± 0.097 b
rapeseed_p1_15	0.621 ± 0.018 d	55.500 ± 0.531 d	14.677 ± 0.090 d
	**svarh**	**senth**	** dvarh**
wheat_p1_15	187.489 ± 3.306 e	1.327 ± 0.053 a	1.792 ± 0.105 a
barley_p1_15	172.468 ± 3.355 b	1.561 ± 0.050 d	2.264 ± 0.106 d
rapeseed_p1_15	180.899 ± 3.392 d	1.433 ± 0.053 b	1.997 ± 0.110 b
	**denth**	**inf1h**	**inf21h**
wheat_p1_15	1.032 ± 0.032 a	− 0.094 ± 0.003 c	0.392 ± 0.012 a
barley_p1_15	1.167 ± 0.027 d	− 0.102 ± 0.003 a	0.441 ± 0.012 d
rapeseed_p1_15	1.097 ± 0.031 b	− 0.096 ± 0.003 b	0.411 ± 0.011 b
	**indnc**	**idmnc**	
wheat_p1_15	0.940 ± 0.003 e	0.977 ± 0.001 e	
barley_p1_15	0.928 ± 0.003 b	0.971 ± 0.001 b	
rapeseed_p1_15	0.934 ± 0.003 d	0.975 ± 0.001 d	

a–e: the differences between mean values with the same letter in columns were statistically insignificant (*p* < 0.05).

**Table 4:** Texture analysis of kernels for selected air stream velocities (20 m s^−1^) and the position of the seed-air tube type I.

	**contr**	**corrm**	**cprom**
wheat_p1_20	1.770 ± 0.106 a	0.448 ± 0.009 a	252.796 ± 24.047 a
barley_p1_20	2.386 ± 0.138 e	0.487 ± 0.007 c	395.250 ± 31.025 e
rapeseed_p1_20	2.039 ± 0.098 bc	0.467 ± 0.008 b	314.162 ± 24.650 bc
	**cshad**	**dissi**	**energ**
wheat_p1_20	− 26.518 ± 2.163 e	0.628 ± 0.032 a	0.444 ± 0.022 e
barley_p1_20	− 38.874 ± 2.588 a	0.811 ± 0.039 e	0.325 ± 0.023 a
rapeseed_p1_20	− 31.964 ± 2.127 cd	0.711 ± 0.029 bc	0.387 ± 0.018 cd
	**entro**	**homom**	**homop**
wheat_p1_20	1.678 ± 0.071 a	0.810 ± 0.009 e	0.812 ± 0.785 e
barley_p1_20	2.085 ± 0.084 e	0.760 ± 0.010 a	0.762 ± 0.728 a
rapeseed_p1_20	1.867 ± 0.063 bc	0.787 ± 0.008 cd	0.788 ± 0.758 cd
	**maxpr**	**sosvh**	**savgh**
wheat_p1_20	0.660 ± 0.017 e	56.605 ± 0.479 e	14.863 ± 0.080 e
barley_p1_20	0.559 ± 0.021 a	53.557 ± 0.701 a	14.344 ± 0.121 a
rapeseed_p1_20	0.614 ± 0.015 cd	55.294 ± 0.471 cd	14.643 ± 0.080 cd
	**svarh**	**senth**	**dvarh**
wheat_p1_20	188.166 ± 3.229 e	1.317 ± 0.052 a	1.770 ± 0.106 a
barley_p1_20	169.094 ± 4.106 a	1.612 ± 0.060 e	2.386 ± 0.138 e
rapeseed_p1_20	179.581 ± 2.962 cd	1.454 ± 0.046 bc	2.039 ± 0.098 bc
	**denth**	**inf1h**	**inf21h**
wheat_p1_20	1.026 ± 0.032 a	− 0.093 ± 0.002 d	0.388 ± 0.011 a
barley_p1_20	1.198 ± 0.033 e	− 0.101 ± 0.002 a	0.446 ± 0.012 e
rapeseed_p1_20	1.109 ± 0.027 bc	− 0.096 ± 0.002 b	0.415 ± 0.010 bc
	**indnc**	**idmnc**	
wheat_p1_20	0.941 ± 0.003 e	0.977 ± 0.001 e	
barley_p1_20	0.924 ± 0.003 a	0.970 ± 0.002 a	
rapeseed_p1_20	0.933 ± 0.003 cd	0.974 ± 0.001 cd	

a–e: the differences between mean values with the same letter in columns were statistically insignificant (*p* < 0.05).

**Table 5:** Texture analysis of kernels for selected air stream velocities (25 m s^−1^) and the position of the seed-air tube type I.

	**contr**	**corrm**	**cprom**
wheat_p1_25	2.020 ± 0.108 b	0.466 ± 0.008 b	311.372 ± 24.739 bc
barley_p1_25	2.344 ± 0.107 e	0.487 ± 0.008 c	384.668 ± 28.773 e
rapeseed_p1_25	2.076 ± 0.110 c	0.467 ± 0.009 b	319.313 ± 27.743 c
	** cshad**	**dissi**	**energ**
wheat_p1_25	− 31.734 ± 2.165 cd	0.703 ± 0.032 b	0.393 ± 0.021 d
barley_p1_25	− 38.047 ± 2.394 a	0.798 ± 0.030 e	0.331 ± 0.018 a
rapeseed_p1_25	− 32.427 ± 2.394 c	0.722 ± 0.032 c	0.380 ± 0.021 c
	**entro**	**homom**	**homop**
wheat_p1_25	1.848 ± 0.072 b	0.789 ± 0.009 d	0.791 ± 0.761 d
barley_p1_25	2.063 ± 0.066 e	0.764 ± 0.008 a	0.765 ± 0.732 a
rapeseed_p1_25	1.889 ± 0.073 c	0.784 ± 0.009 c	0.786 ± 0.755 c
	**maxpr**	**sosvh**	**savgh**
wheat_p1_25	0.619 ± 0.018 d	55.415 ± 0.527 d	14.662 ± 0.089 d
barley_p1_25	0.565 ± 0.017 a	53.725 ± 0.558 a	14.374 ± 0.097 a
rapeseed_p1_25	0.608 ± 0.018 c	55.131 ± 0.554 c	14.615 ± 0.094 c
	**svarh**	**senth**	**dvarh**
wheat_p1_25	180.386 ± 3.348 d	1.440 ± 0.052 b	2.020 ± 0.108 b
barley_p1_25	170.118 ± 3.264 a	1.596 ± 0.048 e	2.344 ± 0.107 e
rapeseed_p1_25	178.563 ± 3.450 c	1.469 ± 0.053 c	2.076 ± 0.110 c
	** denth**	**inf1h**	**inf21h**
wheat_p1_25	1.101 ± 0.031 b	− 0.097 ± 0.002 b	0.413 ± 0.011 bc
barley_p1_25	1.187 ± 0.026 e	− 0.102 ± 0.003 a	0.446 ± 0.011 e
rapeseed_p1_25	1.119 ± 0.030 c	− 0.096 ± 0.003 b	0.417 ± 0.012 c
	**indnc**	**idmnc**	
wheat_p1_25	0.934 ± 0.003 d	0.974 ± 0.001 d	
barley_p1_25	0.926 ± 0.003 a	0.970 ± 0.001 a	
rapeseed_p1_25	0.932 ± 0.003 c	0.974 ± 0.001 c	

a–e: the differences between mean values with the same letter in columns were statistically insignificant (*p* < 0.05).

**Table 6:** Texture analysis of kernels for selected air stream velocities (15 m s^−1^) and the position of the seed-air tube of type II.

	**contr**	**corrm**	**cprom**
rapeseed_p2_15	0.120 ± 0.014 a	0.183 ± 0.016 a	3.982 ± 1.038a
barley_p2_15	0.294 ± 0.076 e	0.249 ± 0.023 e	15.056 ± 5.901e
wheat_p2_15	0.220 ± 0.058 c	0.226 ± 0.022 c	9.772 ± 4.285c
	**cshad**	**dissi**	**energ**
rapeseed_p2_15	− 0.693 ± 0.123 e	0.053 ± 0.006 a	0.940 ± 0.006e
barley_p2_15	− 2.246 ± 0.769 a	0.124 ± 0.030 d	0.866 ± 0.030a
wheat_p2_15	− 1.534 ± 0.561 c	0.094 ± 0.023 c	0.896 ± 0.024c
	**entro**	**homom**	**homop**
rapeseed_p2_15	0.204 ± 0.019 a	0.982 ± 0.002 e	0.980 ± 0.002e
barley_p2_15	0.423 ± 0.087 e	0.959 ± 0.010 a	0.954 ± 0.011a
wheat_p2_15	0.335 ± 0.069 c	0.969 ± 0.007 c	0.965 ± 0.008c
	**maxpr**	**sosvh**	**savgh**
rapeseed_p2_15	0.970 ± 0.003 e	63.330 ± 0.048 e	15.938 ± 0.007e
barley_p2_15	0.930 ± 0.017 a	62.692 ± 0.283 a	15.842 ± 0.043a
wheat_p2_15	0.946 ± 0.013 c	62.965 ± 0.212 c	15.883 ± 0.032c
	**svarh**	**senth**	
rapeseed_p2_15	248.558 ± 0.694 e	0.178 ± 0.016 a	0.120 ± 0.014a
barley_p2_15	240.271 ± 3.357 a	0.358 ± 0.071 e	0.294 ± 0.076e
wheat_p2_15	243.625 ± 2.653 c	0.286 ± 0.057 c	0.220 ± 0.058c
	**denth**	**inf1h**	**inf21h**
rapeseed_p2_15	0.167 ± 0.014 a	− 0.045 ± 0.004 e	0.097 ± 0.007a
barley_p2_15	0.325 ± 0.060 g	− 0.057 ± 0.004 a	0.156 ± 0.021 g
wheat_p2_15	0.263 ± 0.049 e	− 0.053 ± 0.004 c	0.135 ± 0.018e
	**indnc**	**idmnc**	
rapeseed_p2_15	0.995 ± 0.001 e	0.998 ± 0.000 e	
barley_p2_15	0.988 ± 0.003 a	0.996 ± 0.001 a	
wheat_p2_15	0.991 ± 0.002 c	0.997 ± 0.001 c	

a–e: the differences between mean values with the same letter in columns were statistically insignificant (*p* < 0.05).

**Table 7:** Texture analysis of kernels for selected air stream velocities (20 m s^−1^) and the position of the seed-air tube of type II.

	**contr**	**corrm**	**cprom**
rapeseed_p2_20	0.123 ± 0.012 a	0.184 ± 0.014 a	4.082 ± 0.881 a
barley_p2_20	0.182 ± 0.025 b	0.212 ± 0.013 b	7.167 ± 1.727 b
wheat_p2_20	0.214 ± 0.063 c	0.224 ± 0.023 c	9.540 ± 4.604 c
	**cshad**	**dissi**	**energ**
rapeseed_p2_20	− 0.713 ± 0.105 e	0.054 ± 0.005 a	0.939 ± 0.006 e
barley_p2_20	− 1.176 ± 0.225 d	0.079 ± 0.010 b	0.912 ± 0.010 d
wheat_p2_20	− 1.493 ± 0.611 c	0.091 ± 0.025 c	0.899 ± 0.026 c
	**entro**	**homom**	**homop**
rapeseed_p2_20	0.209 ± 0.017 a	0.982 ± 0.002 e	0.979 ± 0.002 e
barley_p2_20	0.289 ± 0.031 b	0.973 ± 0.003 d	0.970 ± 0.004 d
wheat_p2_20	0.326 ± 0.075 c	0.969 ± 0.008 c	0.966 ± 0.009 c
	**maxpr**	**sosvh**	**savgh**
rapeseed_p2_20	0.969 ± 0.003 e	63.318 ± 0.044 e	15.936 ± 0.007 e
barley_p2_20	0.955 ± 0.006 d	63.104 ± 0.089 d	15.904 ± 0.013 d
wheat_p2_20	0.948 ± 0.014 c	62.989 ± 0.231 c	15.887 ± 0.035 c
	**svarh**	**senth**	**dvarh**
rapeseed_p2_20	248.382 ± 0.636 e	0.182 ± 0.014 a	0.123 ± 0.012 a
barley_p2_20	245.388 ± 1.161 d	0.248 ± 0.025 b	0.182 ± 0.025 b
wheat_p2_20	243.950 ± 2.883 c	0.279 ± 0.062 c	0.214 ± 0.063 c
	**denth**	**inf1h**	**inf21h**
rapeseed_p2_20	0.171 ± 0.013 bc	− 0.045 ± 0.003 e	0.098 ± 0.006 bc
barley_p2_20	0.230 ± 0.022 d	− 0.051 ± 0.003 d	0.122 ± 0.009 d
wheat_p2_20	0.257 ± 0.053 e	− 0.053 ± 0.005 c	0.133 ± 0.020 e
	**indnc**	**idmnc**	
rapeseed_p2_20	0.995 ± 0.001 e	0.998 ± 0.000 e	
barley_p2_20	0.992 ± 0.001 d	0.998 ± 0.000 d	
wheat_p2_20	0.991 ± 0.002 c	0.997 ± 0.001 c	

a–e: the differences between mean values with the same letter in columns were statistically insignificant (*p* < 0.05).

**Table 8:** Texture analysis of kernels for selected air stream velocities (25 m s^−1^) and the position of the seed-air tube of type II.

	**contr**	**corrm**	**cprom**
rapeseed_p2_25	0.137 ± 0.012 a	0.190 ± 0.013 a	4.615 ± 0.844 a
barley_p2_25	0.257 ± 0.098 d	0.238 ± 0.030 d	12.741 ± 7.658 d
wheat_p2_25	0.212 ± 0.025 c	0.224 ± 0.016 c	9.076 ± 2.201 c
	** cshad**	**dissi**	**energ**
rapeseed_p2_25	− 0.807 ± 0.104 e	0.060 ± 0.005 a	0.933 ± 0.005 e
barley_p2_25	− 1.921 ± 0.995 b	0.109 ± 0.039 e	0.882 ± 0.039 b
wheat_p2_25	− 1.444 ± 0.267 c	0.091 ± 0.010 c	0.899 ± 0.011 c
	**entro**	**homom**	**homop**
rapeseed_p2_25	0.228 ± 0.016 a	0.980 ± 0.002 e	0.977 ± 0.002 e
barley_p2_25	0.377 ± 0.113 d	0.964 ± 0.012 b	0.959 ± 0.014 b
wheat_p2_25	0.326 ± 0.032 c	0.970 ± 0.003 c	0.966 ± 0.004 c
	**maxpr**	**sosvh**	**savgh**
rapeseed_p2_25	0.966 ± 0.003 e	63.270 ± 0.042 e	15.929 ± 0.006 e
barley_p2_25	0.939 ± 0.021 b	62.827 ± 0.362 b	15.862 ± 0.055 b
wheat_p2_25	0.948 ± 0.006 c	62.996 ± 0.094 c	15.888 ± 0.014 c
	**svarh**	**senth**	**dvarh**
rapeseed_p2_25	247.671 ± 0.603 e	0.198 ± 0.013 a	0.137 ± 0.012 a
barley_p2_25	242.002 ± 4.342 b	0.320 ± 0.092 d	0.257 ± 0.098 d
wheat_p2_25	243.967 ± 1.207 c	0.279 ± 0.026 c	0.212 ± 0.025 c
	** denth**	**inf1h**	**inf21h**
rapeseed_p2_25	0.186 ± 0.012 c	− 0.047 ± 0.003 e	0.104 ± 0.006 c
barley_p2_25	0.292 ± 0.078 f	− 0.056 ± 0.005 b	0.145 ± 0.028 f
wheat_p2_25	0.258 ± 0.023 e	− 0.053 ± 0.003 c	0.133 ± 0.010 e
	**indnc**	**idmnc**	
rapeseed_p2_25	0.994 ± 0.000 e	0.998 ± 0.000 e	
barley_p2_25	0.989 ± 0.004 b	0.997 ± 0.001 b	
wheat_p2_25	0.991 ± 0.001 c	0.997 ± 0.000 c	

a–e: the differences between mean values with the same letter in columns were statistically insignificant (*p* < 0.05).

Similarity measured for the seed-air tube of type II occurred for variables: contr, corrm, cprom, dissi, entro, senth and dvarh between research classes in rapeseed when the air stream velocity was set at 15 m s^-1^ (Table 6), 20 m s^-1^ (Table 7), 25 m s^−1^ (Table 8). In the case of variables cshad, energ, homom, homop, maxpr, sosvh, savgh, svarh, indnc and idmnc it was observed that the most significant research group was barely with the type II tube, for air stream velocity equal to 15 m s^−1^. A statistical comparison of texture descriptors (Tables 6, 7, 8) for the seed-air tube of type II, made it possible to find similarities between fine-grained kernels of rapeseed and medium size barley kernels.

Taking into consideration the seed-air tubes of type I and II it can be concluded that, according to the texture descriptors, the biggest similarity occurred in the research group including medium size kernels i.e. barley. It is worth noting that the air stream velocity has the major influence on the image object recognition. Comparing the results of the research, for the needs of which artificial neural networks were used to identify the sowing material (triticale) contamination and taking into consideration velocity of the sowing material, it was possible to recognize the kernel contamination classes on the basis of an image^37^. Analyzing the degree of difficulty in fast identification of wheat, barley and rape kernels, like in the analysis of triticale contamination^37^ , velocity variants and different devices supporting transport of seeds were taken into consideration. Apart from kernel variants, the techniques that were used supported by MLPN allowed to obtain satisfying results characterized by high coefficient of classification, especially for sowing velocity equal to 20 m s^−1^. ANN created during the research can be a useful device supporting measurement of velocity of kernels transported pneumatically.

Other studies have proven that the machine learning technique allows to evaluate effectively filamentous fungus affected fine-grained kernels (rapeseed) by using the microscopic technique^48^. Thanks to further and deeper analyses it was noticed that high classification effectiveness was also possible for various food products and different grain varieties^28,48^. Properties of kernels properly determined on the basis of a bitmat including: thousand grain mass, geometrical diameter, coefficient of spherical shape, surface area, porosity, color and texture, enable fast and non-invasive assessment of the grain class for a given velocity of sowing.

## Conslusions

MLPNs capable of noninvasive recognition of wheat, barley and rape kernels were devised based on texture descriptors. The tests were performed using the author’s original test stand, which was supposed to emulate real conditions of the sowing process. Only 2 structural solutions of transporting kernels via a seed-air tube were tested.

It was found that an increase in air stream velocity had a negative impact on the effectiveness in recognizing individual classes of kernels transported via seed-air tubes with the use of structures I and II. It turned out that, the most optimal air stream velocity for kernels transported via the pipe (for solution I) was equal to15 m s^−1^. It should be added that the most optimal MLP was MLP-p1_15, which reached RMSE value of 0.060 and classification accuracy coefficient at the level of 0.94. In the case of II type air and seed pipe, the best MLP turned out to be MLP-p2_20, which reached the best result for RMSE value of 0.050.

An analysis of variations allowed to group effectively all research classes. It also allowed to determine the impact of texture variables for individual grain varieties including the air stream velocity and the type of air and seed pipe. In the future, the obtained results can be used for further research on the author’s original system for detecting and measuring the amount of sowing material.
